# ProProtein: A platform for fully automated identification of 3D structure fluctuations in MD simulation trajectories

**DOI:** 10.1371/journal.pone.0329314

**Published:** 2025-08-06

**Authors:** Krzysztof Mularski, Dawid Makalowski, Marcin Okonek, Daria Glebowska, Mateusz Swiercz, Karol Kamel, Jacek Blazewicz, Maciej Antczak, Aleksandra Swiercz

**Affiliations:** 1 Institute of Computing Science, Poznan University of Technology, Poznan, Poland; 2 Institute of Bioorganic Chemistry, Polish Academy of Sciences, Poznan, Poland; AlloTec Bio, UNITED STATES OF AMERICA

## Abstract

The main goal of Molecular Dynamics (MD) is a simulation of a physical system motions in a fixed time period. This technique allows users to observe the dynamic evolution of the system but requires advanced force fields and is computationally intensive. Furthermore, finding desirable features in the results obtained is usually a time-consuming task. This motivates the need to implement a tool that visualizes the most flexible protein fragments. ProProtein platform is a sophisticated web server where, with a single click, the user can set up, configure, and run an MD simulation of the 3D structure of the peptide/protein. We perform the simulation using an open-source software suite developed for high-performance molecular dynamics named Gromacs. The resulting MD trajectory is then automatically analyzed within the dedicated heuristic algorithm to identify 3D fragments characterized by high instability in the context of the given (input) structure. These high-fluctuation substructures are presented next to the user with the Mol* package. They are visualized in colors on each frame covered by the considered trajectory. This tool can easily support the evaluation of the reliability of protein 3D structure predictions obtained computationally. The ProProtein platform is free and open to all users. It is publicly available at: https://proprotein.cs.put.poznan.pl/.

## Introduction

Molecular Dynamics (MD) is a preferred method for studying complex systems across various fields of modern science, including materials science, chemical physics, biophysics, and structural biology. MD simulations enable the modeling of the physical movements and interactions of the 3D structure of a molecule, resulting in a trajectory that depicts the dynamic properties and evolution of a system over time. In the 21st century, the remarkable advancements in computational power, the utilization of graphics processing units (GPUs) for extensive parallel computation, and the deployment of supercomputers built on application-specific integrated circuits (ASICs) have allowed scientists to examine systems of unprecedented sizes [1, 2] and timescales [3]. This computational progress is particularly significant in biophysics and structural biology, where the size, complexity, and simulation time required often present major challenges. Also the amount of data is growing so quickly that its reliable analysis requires the use of various computational and algorithmic techniques [4–7].

Numerous software packages can perform MD simulations, Gromacs [8] and NAMD [9] being among the most widely used. These packages employ parameterized force fields for the simulation of biological components such as proteins, nucleic acids, and lipids. They also support GPU processing and are available for non-commercial applications. Running an MD simulation and obtaining a trajectory is just the initial, albeit most challenging, step due to the computational cost and time involved. Although MD trajectories contain valuable information, extracting significant insights from them can be difficult.

Several web-based platforms and tools have emerged to facilitate MD simulations, including MDWeb [10], WebGRO [11], Making it Rain [12], and Visual Dynamics [13]. These platforms offer various features and mostly support the Gromacs simulation package. MDWeb, for instance, is highly comprehensive, supports multiple MD packages such as Amber [14, 15] and NAMD, and provides services for setting up, running simulations, and analyzing the flexibility in the resultant MD trajectories. The Making it Rain tool leverages easy-customizable Jupyter notebooks, allowing users to perform molecular system simulations using the OpenMM toolkit on the Google Colab framework.

The Visual Dynamics and Making It Rain platforms fully automatically support protein-ligand simulations. WebGro uses a limited set of GROMOS force-field parameters, and MDWeb does not support a simulation scenario of this kind. All of the tools considered, except FlexServ provided via MDWeb, do not allow for further identification and visualization of high-fluctuation 3D fragments. Moreover, all web-accessible platforms require users to register to maintain a permanent workspace. Only MDWeb supports anonymous user projects, but they are removed after periods of inactivity, thus limiting practical use.

In contrast, the proposed platform is free and open to all users and supports task processing submitted by anonymous users. The task results page is provided via a specific URL, which can be optionally sent to the user’s email (limit to 10 tasks per user) on demand. The results page remains accessible for 14 days after the task is completed. ProProtein enables users to run the MD simulation of a single peptide/protein-solvated molecule using Gromacs (2021.5), to visualize resultant trajectories, and to conduct fully automated or user-assisted analysis of the stability/flexibility of 3D fragments.

## Materials and methods

**Fig 1:**
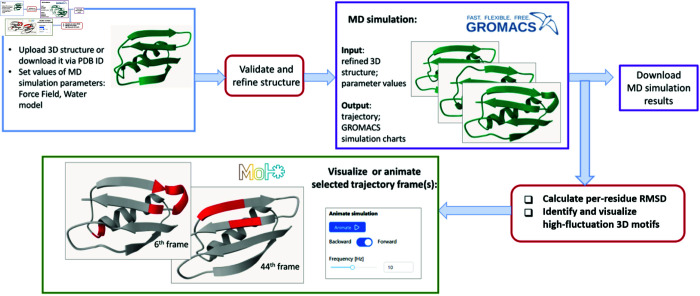
Workflow of the ProProtein platform.

The ProProtein platform’s workflow comprises four main steps: (i) uploading and verifying the 3D structure of the given protein, (ii) running molecular dynamics (MD) simulations using the Gromacs MD simulation suite, (iii) identifying the most significant fluctuations within the 3D structure, and (iv) visualizing these fluctuations in the context of the MD simulation trajectory (Fig 1).

The MD simulation performed by the Gromacs suite is the most computationally intensive step in this workflow. Once the simulation is completed, a dedicated heuristic algorithm identifies significant fluctuations in the 3D structure trajectory of the protein, highlighting the regions with the highest degrees of flexibility.

**Fig 2:**
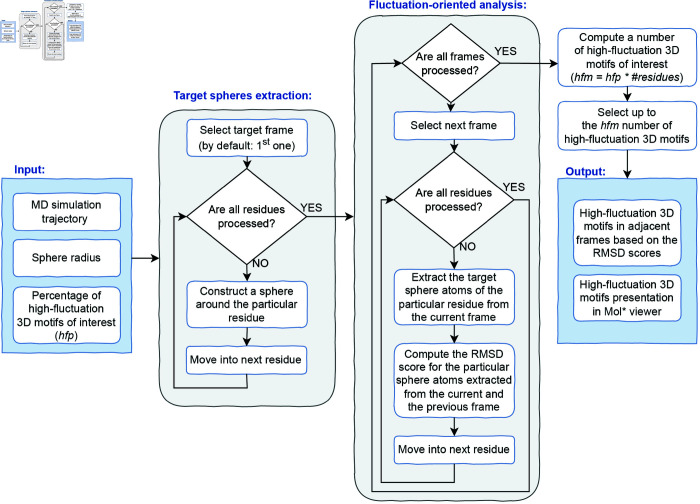
A heuristic for identification of high-fluctuation 3D motifs of interest.

As shown in Fig 2, the heuristic for the identification of high-fluctuation 3D motifs initiates with the MD simulation trajectory, where each frame of the trajectory is stored in PDB format. The spatial neighborhood around each residue is constructed using a user-defined radius of the sphere. The high-fluctuation motifs are determined by calculating the Root Mean Square Deviation (RMSD) scores of the atoms belonging to the particular sphere in the two subsequent frames. The user can define the percentage of the motifs with the highest RMSD scores. The number of motifs of interest results from the product of the number of residues and the number of frames decremented by one.

In the first phase, for each residue ($C_{\alpha }$ atom) along the chain of the target frame (by default, the first frame), a sphere is constructed that includes all atoms located within a spatial proximity threshold. The spatial proximity parameter, *d*, defines the Euclidean distance within which atoms are considered in spatial contact, adjustable from 2 to 8 Å during task setup.

Next, we compare subsequent frames in the MD trajectory. For each residue in the current frame, we extract the corresponding atoms within the sphere, identified previously in the target frame, allowing for a dynamic view of fluctuations within the same neighborhood over time. An RMSD score is computed for each residue’s sphere in the current frame relative to the previous frame, quantifying the degree of fluctuation.

The presented approach differs from traditional methods such as RMSF or tRMSF, which measure atomic fluctuations relative to their mean positions across the trajectory. Instead, we use RMSD to quantify frame-to-frame deviations of spatially defined local neighborhoods (spheres) around each residue, thus highlighting regions of structural variability. The concept of spatial neighborhoods, inspired by the SphereGrinder tool [16], is used here to track motif’ fluctuations over time. The tool mentioned performs the local-to-global model quality assessment.

After calculating the RMSD scores, we select a specified number of high-fluctuation 3D motifs of interest (*hfm*) for detailed analysis. These motifs are visualized in 3D and highlighted in color using the Mol* viewer, enhancing the interpretability of spatial fluctuations within functionally significant regions.

This approach for identifying high-fluctuation 3D motifs provides deeper insights into the molecular dynamics of the analyzed structure, as it considers not only individual residues but also their spatial neighborhoods, which may contain critical long-range interactions.

The ProProtein platform is built using a multilayer architecture. The user interface is developed in REACT to be responsive and compatible with web browsers and mobile devices. The backend component, implemented in TypeScript operated on the Express framework, incorporates a NoSQL MongoDB database and employs the toad-scheduler package to manage Gromacs-based MD simulations. Additionally, we utilize C++ and Python scripts to efficiently compute RMSD scores and convert PostScript files to PNG images for visual output.

## Results

### ProProtein platform

First, a user uploads a 3D protein structure in PDB format. Alternatively, the user can submit an example structure provided by the platform or download the structure directly from the RCSB PDB database [17] using a specified PDB ID. The uploaded file is then validated by the platform. During this process, all nonprotein chains, ligands, and ions are automatically discarded. In addition, any amino acids missing some atom coordinates are rejected, and any format-related abnormalities are reported to the user. The complete log file of the protein validation process is reported to user as an output file.

During task submission, users can configure MD simulation parameters, such as force field, water model, simulation length, and saving step. Moreover, the option ’high-fluctuation 3D motifs of interest’ allows users to set the percentage of the most flexible fragments colored red based on the RMSD values. Finally, the ’sphere radius’ parameter allows users to set the interatomic distance threshold computed between $C_{\alpha }$ atoms that cannot be exceeded by residues located close to each other in 3D. Before running the simulation, the user can visualize the uploaded/solvated structure with the chosen parameters.

After submitting a job, the user is redirected to a page presenting the task results and can opt to receive an email notification when the submission is completed, if an email address was provided in the input form. The task results page summarizes the MD simulation configuration parameters and the current processing status of the task. In addition, the values of all input parameters are reported. Other features on the results page remain disabled until the job is finished. The input 3D structure of the loaded protein is visualized in the Mol* viewer [18] showing all high-fluctuation motifs in red. The user can also switch between the particular frames considered in the current trajectory. Finally, the user can download all the simulation results in one step via the ’All’ button or each component result, e.g., the trajectory PDB file, independently.

In addition to visualization of the most flexible fragments, users can download various outputs from the Gromacs simulation suite. These outputs include the output trajectory and plots for potential energy, temperature, density, pressure, RMSF (Root Mean Square Fluctuation), and radius of gyration. To enable users to analyze flexible fragments in detail outside the system, we provide an output CSV file that includes the RMSD score matrix presenting scores for all residue neighborhoods in all frames considered.

If the users want to change the simulation options without re-uploading the input 3D structure, they can go to the bottom of the webpage and resubmit the job with modified parameters.

MD simulation is a time consuming process and the overall time depends on the length of the protein structure and the ’simulation length’ parameter.

### Case studies

**Fig 3:**
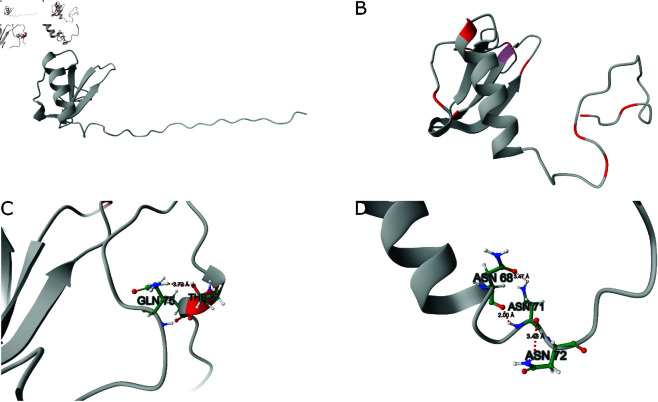
Example MD simulation results. Panel A shows the human lymphotactin protein model with an unfolded C-terminal fragment, representing the starting structure of the MD production run. Panel B presents the formation of a loop in the C-terminal fragment at 5 ns (high-fluctuation 3D motifs are depicted in red color). Panel C shows the HB interactions in the folded C-terminal fragment at 10 ns. Panel D shows proximal residues of the C-terminal fragment at 15 ns, where an altered hydrogen bonding network stabilizes the bent C-terminal conformation.

A specialized MD simulation was performed to demonstrate the capabilities of the platform. The system of interest is human lymphotactin, a 93-residue protein belonging to the cytokine family [19] (Fig 3A). This protein plays a crucial role in the migration of white blood cells, particularly in attracting T lymphocytes. The protein molecule (PDB ID: 1J8I) was prepared for simulation using UCSF Chimera software [20]. The Amber99SB-ILDN force filed [21] was applied, and the molecular system was solvated in a TIP3P water model, neutralized, and supplemented with 0.1 M NaCl to mimic intercellular conditions. After energy minimization, the system underwent a 100-ps equilibration run, followed by a 15-ns production run at 298K, with configurations (frames) recorded every five ps.

Analysis of the resulting trajectory showed that the long, unstructured C-terminal fragment (26 residues) underwent folding (Fig 3B), stabilized initially by hydrogen bonds, particularly side-chain interactions between Gln75 and Thr87 (Fig 3C). Over the simulation course, this hairpin-like structure began to unfold, giving rise to a more stable conformation (Fig 3D). The interaction between Asn68 and Asn71, facilitated by both backbone and side-chain hydrogen bonding, formed a structural scaffold, with additional stabilization provided by Asn72 via amide carbonyl group hydrogen bonding.

**Fig 4:**
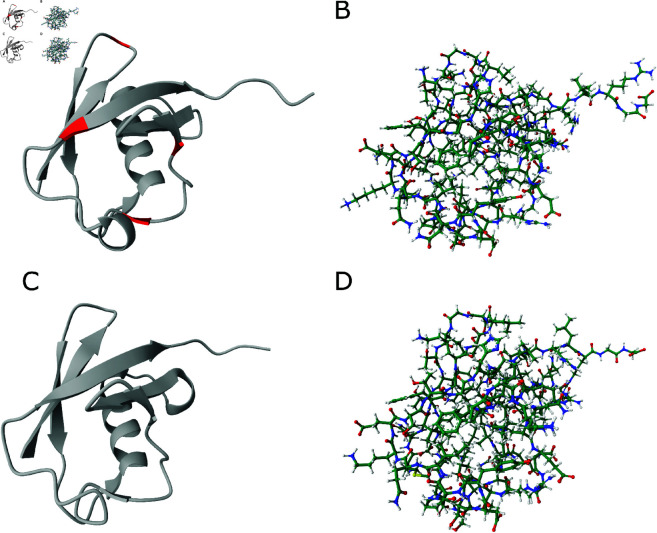
Example MD simulation results. Panels A and B show the human ubiquitin protein model at the five ns MD production run. Panels C and D show the ubiquitin model at the 15 ns of the simulation run. High-fluctuation 3D motifs are shown in red color (panel A).

For the second example, we selected ubiquitin (PDB ID: 1UBQ) [22], a small yet structurally stable protein present in all eukaryotic cells. Ubiquitin is associated with various cellular functions, such as regulating protein interactions, marking proteins for degradation, or modulating protein activity. The ubiquitin model was prepared for simulation following the same protocol as the lymphotactin model and applying the same simulation scheme. As shown in Fig 4A–4D, ubiquitin remained structurally stable throughout the simulation, with only a few residues exhibiting minor flexibility, while the remainder of the protein maintained its functional conformation, stabilized by a dense network of hydrogen bonds.

## Conclusion

In molecular dynamics, there is a growing need for tools that support researchers in an efficient and detailed analysis of the obtained results. While several web servers, such as Making it Rain, already provide in-depth analysis tools to assess tertiary structure flexibility. However, there remains a demand for solutions that prioritize simplicity and accessibility, particularly in educational contexts. The ProProtein platform addresses this need by offering a user-friendly interface that enables users to easily experiment with the most commonly used dynamics simulation parameters, define the expected size of the 3D motifs of interest (the sphere radius), specify the percentage of highly-fluctuated motifs under interest, interactively visualize the detected motifs. Additionally, all result files – including the validation log and summary of identified high-fluctuation regions – are available for download, facilitating further analysis or post-processing.

To make the tool widely accessible, ProProtein enables custom molecular dynamics simulations without requiring registration, using the Gromacs software suite. Once a task is completed, users are presented with a comprehensive results page that allows them to explore the full simulation trajectory and identify fluctuating fragments with ease.

In the future, we plan to enhance the platform with more advanced algorithms that allow users to detect frame pairs in the given trajectory where 3D substructures differ the most. Moreover, we also intend to incorporate GPU-based computation to improve the performance of 3D structure comparison.
